# MaxEnt-based evaluation of climate change effects on the habitat suitability of *Magnolia officinalis* in China

**DOI:** 10.3389/fpls.2025.1601585

**Published:** 2025-07-08

**Authors:** Jun Ren, Suhang Li, Yawen Zhang, Qiong Yang, Jiaojiao Liu, Jing Fan, Yangzhou Xiang

**Affiliations:** ^1^ School of Geography and Resources, Guizhou Education University, Guiyang, China; ^2^ School of Pharmacy, Lanzhou University, Lanzhou, China

**Keywords:** *Magnolia officinalis*, medicinal tree species, MAXENT model, potential distribution, shared socioeconomic pathway

## Abstract

This study aimed to assess the impact of climate change on the potential distribution of the endangered medicinal plant *M. officinalis* in China. We sought to identify key bioclimatic variables influencing its distribution, predict current and future suitable habitats, and evaluate shifts in these habitats under different climate scenarios. We constructed a dataset comprising 405 distribution records of *M. officinalis* and 9 major environmental factors. The MaxEnt model, integrated with GIS software, was employed to predict the potential distribution under current (1970-2000) and future periods (2050s, 2070s, and 2090s). Model optimization was conducted using the ENMeval package to adjust regularization multiplier and feature combination parameters, ensuring enhanced predictive accuracy. The optimized MaxEnt model demonstrated high predictive precision with an AUC value of 0.917. The minimum temperature of the coldest month, mean diurnal range, and annual precipitation were identified as the key environmental variables influencing *M. officinalis* distribution, with contribution rates of 72.7%, 11.6%, and 4.2%, respectively. The suitable habitat was predicted to expand by 2050s under the SSP1-2.6 scenario but showed a reduction in highly suitable areas under more severe scenarios like SSP5-8.5. Centroid shift analyses indicated a northwestward migration of suitable habitats. These results from this study suggest that climate change poses significant risks to the distribution of *M. officinalis*, with potential shifts in both the extent and quality of suitable habitats. Our findings highlight the importance of considering climate change projections in conservation planning and underscore the need for adaptive strategies to ensure the sustainability of this medicinally valuable species. The study provides a scientific basis for the conservation and sustainable use of *M. officinalis* in the context of climate change.

## 1 Introduction

Plants sustain human life in numerous ways, forming the foundation of the food chain and providing a multitude of essential resources and services (Pironon et al., 2024). Understanding species distribution patterns is crucial for the sustainable conservation and management of plant resources. This not only helps to protect biodiversity and ensure the health and stability of ecosystems (Randin et al., 2020), but also has a direct impact on agricultural production, the preservation of traditional knowledge, and the human well-being (Pecl et al., 2017; Mi et al., 2021). The geographical distribution of plant species is shaped by a variety of factors, encompassing the inherent physiological and genetic traits of the plants (Laughlin et al., 2021), anthropogenic influences (Xu et al., 2019), as well as other environmental conditions, including temperature and humidity as climatic factors (Coelho et al., 2023). An escalating number of investigations reveal that the frequency of extreme climate events has risen in numerous regions as a consequence of global warming (Diffenbaugh et al., 2017; Zhang and Zhou, 2020). Furthermore, future extreme climate events are likely to become more intense and frequent in high-emission scenarios (Fischer et al., 2021; Fowler et al., 2021). Therefore, there is no doubt that plant species faces significant risks from extreme climate events (Manes et al., 2021; Trew and Maclean, 2021; Singh et al., 2023). Accordingly, evaluating the effects of climate change on the distribution of plant species is essential for deciphering the relationship between climatic conditions and their geographical distribution, which in turn offers a scientific foundation for the preservation of biodiversity and the deliberate introduction of plants for agricultural purposes.

Climate is one of the main determinants delimiting geographical distribution of plant species on large scales (Ferrarini et al., 2019). There is considerable research demonstrating that climate change leads to range expansion or retraction in plant species distributions (Thuiller et al., 2005). To assess the vulnerability of plant species under a rapidly changing climate, species distribution modeling (SDM) can be employed to predict species climate niches and project their potential future range shifts (Huntley et al., 1995; Pearson and Dawson, 2003). They use the locations where species have been found and information about the environmental data, including factors like climate, topography, and soil. These models help us understand how species are linked to their environment. They also calculate the chances of species living in certain areas based on the environment’s suitability (Elith and Leathwick, 2009; A. Lee-Yaw et al., 2022; Hui, 2023). With the ongoing development of theories in mathematical statistics and ecology, an increasing variety of models for predicting species distribution is being developed, encompassing Biological Climatic Model (BIOCLIM) (Beaumont et al., 2005), General Additive Model (GAM) (Hijmans and Graham, 2006), Generalized Linear Model (GLM) (Guisan et al., 2002), Mechanistic Niche Model (CLIMEX) (Kriticos et al., 2012), Maximum Entropy Model (MaxEnt) (Phillips et al., 2006). Among them, the MaxEnt model, which is founded on the principle of maximum entropy (Xu et al., 2018), offers numerous benefits in species distribution modeling, such as flexibility in sample size requirements, sensitivity to environmental variables, strong model interpretability, user-friendliness, as well as efficiency in computational time (Phillips et al., 2006; Kaky et al., 2020; Khan et al., 2022).

In the field of global change and biogeography research, the response of vegetation to climate change has always been a core focus (Bellard et al., 2012). Climate, as a key environmental factor influencing species and vegetation distribution at both regional and global scales, has profound and far-reaching effects on biodiversity and species range (Hamann and Wang, 2006). Future climate change can lead to shifts in the distribution and abundance of species (Thomas et al., 2004; Ehrlén and Morris, 2015), range shifts (Chen et al., 2011; Bellard et al., 2012), phenological changes (Merilä and Hendry, 2014; Cuena-Lombraña et al., 2018), and physiological trait changes (Fois et al., 2018). Over the past decades, there has been a growing focus on examining how climate change affects the potential distribution areas of plant species through MaxEnt models. For example, utilizing the MaxEnt method, Khanum et al. (2013) projected the potential climatic habitats of three medicinally important Asclepiad species indigenous to Pakistan, and found that projected climate change scenarios could moderately to significantly affect their geographic distributions of three species. The MaxEnt model was employed to forecast the potential distribution of the endangered medicinal plant (*Homonoia riparia* Lour.) in Yunnan, China, by incorporating topographic and bioclimatic variables (Yi et al., 2016b). The potential suitable areas of an important economic and medicinal tree (*Litsea cubeba* (Lour.) Pers.) in China for current and future climates are predicted using an optimized MaxEnt model, identifying key environmental factors as precipitation of the driest quarter, annual precipitation, temperature annual range and minimum temperature of the coldest month (Shi et al., 2023). With the help of MaxEnt model, Wang et al. (2024a) predicts 21st-century habitat suitability for a high economic and medicinal species (*Chionanthus retusus* Lindl. & Paxton), evaluates climate change effects, and pinpoints critical areas for conservation. Recently, Zhang et al. (2024b) adopted MaxEnt model to evaluate the ecological quality of the medicinal tree species (*Eucommia ulmoides* Oliv.) in China, and emphasizes the pivotal role of climatic factors in shaping its geographic distribution and projects potential habitat shifts in the context of future climate change scenarios. In summary, the MaxEnt model has been widely used to study the effects of climate change on the potential distribution of plant species, forecasting shifts in the geography of numerous medicinal plants, and highlighting the pivotal role of climatic factors in shaping distribution patterns and predicting future habitat changes. While comparative studies between SDMs are valuable, recent meta-analyses have demonstrated that optimized MaxEnt models consistently outperform other presence-only methods across diverse taxa and regions (Elith et al., 2011; Merow et al., 2013). For *M. officinalis* specifically, the complex topography and heterogeneous climate of its distribution range favour MaxEnt’s machine-learning approach over simpler envelope models like BIOCLIM.

*Magnolia officinalis* Rehd. et Wils (*M. officinalis*), belonging to the Magnoliaceae family and the Houpoea genus, is a deciduous tree that is unique to China and is classified as a nationally protected endangered medicinal plant of the second level (Tan et al., 2019). In recent years, significant advancements have been made in the study of *M. officinalis* with the development of modern pharmacology and chemical analysis techniques (Luo et al., 2019; Niu et al., 2021). A variety of active components, including alkaloids (Guo et al., 2019), flavonoids, terpenoid compounds (Sun et al., 2024), and volatile oils (Liu et al., 2024b), have been identified from the bark or leaves of *M. officinalis* using techniques such as high-performance liquid chromatography (Yi et al., 2016a), gas chromatography-mass spectrometry (Qi et al., 2024), and ultra-performance liquid chromatography-mass spectrometry (Huang et al., 2023). These constituents have demonstrated multiple biological activities, such as anti-inflammatory, antioxidant, antitumor, and neuroprotective effects (Youn et al., 2013; Hao et al., 2024). Furthermore, the mechanisms of action of these active components at the molecular level are being explored using molecular biological techniques, including the regulation of inflammatory mediator release, inhibition of tumor cell proliferation, and induction of apoptosis (Zhong et al., 2022; Li et al., 2023). Concurrently, attention has been given to the conservation of the genetic diversity and germplasm resources of *M. officinalis* to ensure the sustainable use of this valuable medicinal material (He et al., 2009; Yang et al., 2020). However, to our best knowledge, studies examining the impact of climate change on the potential distribution of *M. officinalis* have not been documented.

To provide a scientific basis for the survey of germplasm resources, the protection of wild resources, the domestication of artificial introduction, the construction of artificial forests, and the sustainable development of the industry for *M. officinalis* in China, it is particularly crucial to accurately identify its potential climatic suitability zones and ecological environmental impact factors in China. To achieve these goals, we used ecological niche modelling with MaxEnt and bioclimatic variables from WorldClim to estimate current and future distributions of *M. officinalis* under different climate scenarios. The objectives of this study are: (1) to identify the key bioclimatic variables that contribute to predicting the potential distribution ranges of *M. officinalis*; (2) to predict the distribution pattern of the potential suitable areas for *M. officinalis* under current and future climatic conditions, and to classify them into different suitability levels; (3) to quantify the changes in the geographical ranges and spatial patterns of suitable habitats for *M. officinalis* under projected future climate conditions.

## 2 Materials and methods

### 2.1 Collection and processing of M. officinalis occurrence records

**Figure 1:**
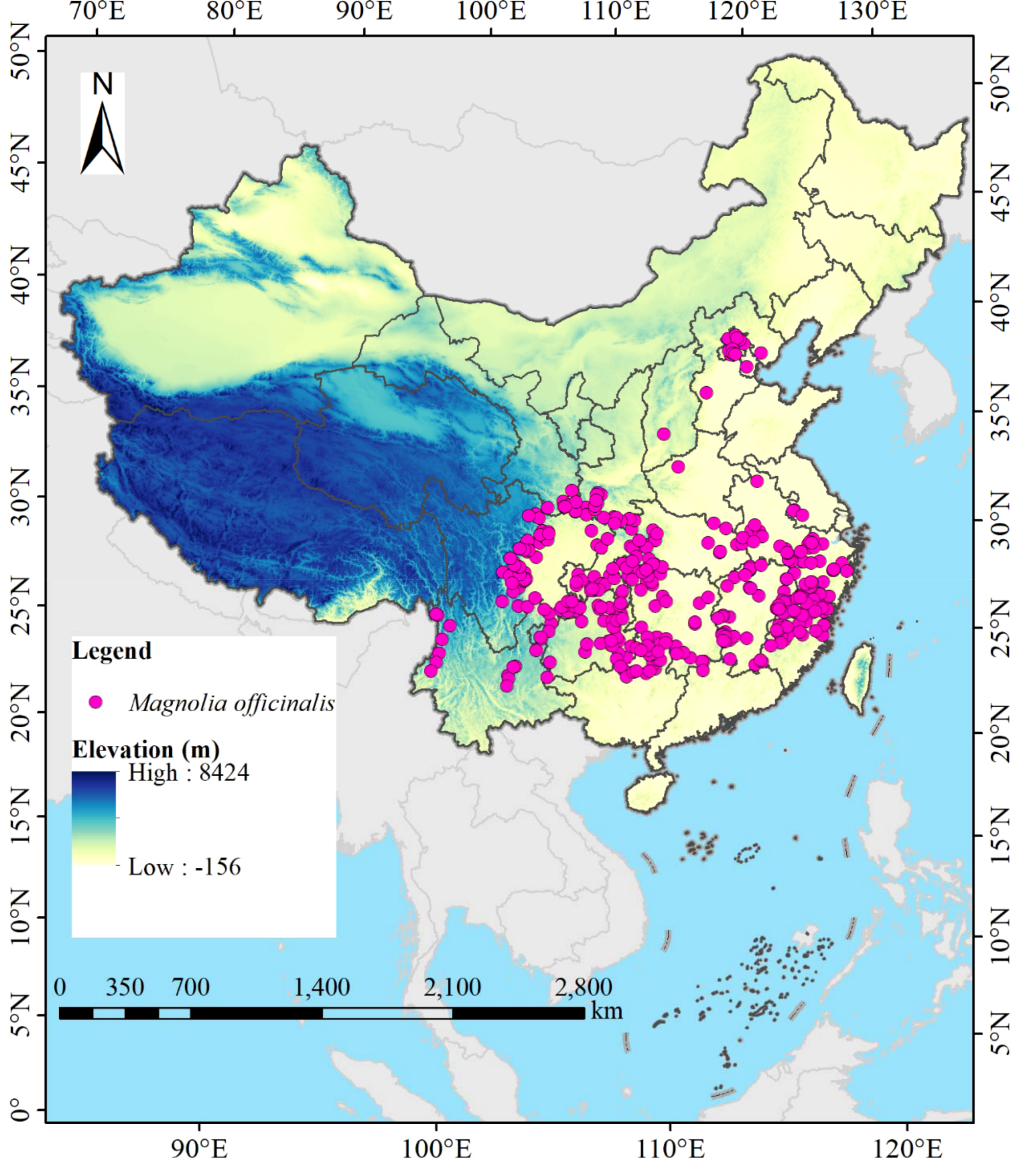
Location of 405 distribution points of *M. officinalis* in China.

The distribution data of *M. officinalis* in China were sourced from the National Specimen Information Infrastructure (NSII, http://www.nsii.org.cn/, accessed on July 12, 2024), the Chinese Virtual Herbarium (CVH, http://www.cvh.org.cn/, accessed on July 15, 2024), the Global Biodiversity Information Facility (GBIF, https://www.gbif.org, accessed on July 16, 2024), and scientific literature published on China National Knowledge Infrastructure (CNKI, https://www.cnki.net/, accessed on July 18, 2024) and Web of Science (WOS, https://clarivate.com.cn/, accessed on July 22, 2024), totaling 429 distribution points for the species. We now specify that for distribution points with clear location reports but lacking latitude and longitude data, we used the website to convert place names into coordinates, following Chapman and Wieczorek (2006) for spatial uncertainty quantification. To eliminate the impact of spatial autocorrelation of distribution points on model prediction accuracy (Halvorsen et al., 2016), a grid file with a resolution of 2.5′×2.5′ was added in ArcGIS 10.8, and *M. officinalis* distribution points were manually selected to ensure that only one point closest to the center of each grid was retained. To minimize temporal bias, we restricted our analysis to occurrence records from 1970-2020, aligning with the temporal scope of our climate data. Historical herbarium specimens predating 1970 (n=24) were excluded to ensure consistency with contemporary climate conditions. For spatial bias correction, we applied spatial thinning using a 5 km buffer to reduce sampling bias in over-represented areas. Ultimately, 405 valid data of *M. officinalis* were obtained (**Figure 1**). The species name and the latitude and longitude information of the distribution points were entered into Excel 2016 software in.xls format. To facilitate subsequent data analysis, the file format recording the distribution information of *M. officinalis* was converted from.xls to.csv format.

### 2.2 Data collection and processing of environmental variables

The Bioclimatic variables used in this study to predict the potential distribution of *M. officinalis* under current (1970-2000) climate conditions were derived from the WorldClim database (https://www.worldclim.org/data/worldclim21.html, accessed on January 12, 2024), with a spatial resolution of 2.5′×2.5′ (Fick and Hijmans, 2017). To meet the data format requirements of the MaxEnt software, the climate data in Tiff format were converted to ASCII format using ArcGIS 10.8 software. Environmental data at a spatial resolution of 2.5′×2.5′ from the BCC-CSM2-MR model, known for accurately simulating climate across China, were used to predict the changes in the suitable habitat of *M. officinalis* (Wu et al., 2019). Additionally, we selected future environmental data under three greenhouse gas emission scenarios represented by Shared Socioeconomic Pathways (SSP). These scenarios include: a low concentration of greenhouse gas emissions (SSP1-2.6), a medium-high concentration of greenhouse gas emissions (SSP3-7.0), and the highest concentration of greenhouse gas emissions (SSP5-8.5), representing the achievement of forcing levels of 2.6, 7.0, and 8.5 W m^−2^ by 2100, respectively (He et al., 2023). These scenarios correspond to three periods: the 2050s (2041-2060), the 2070s (2061-2080), and the 2090s (2081-2100).

**Figure 2:**
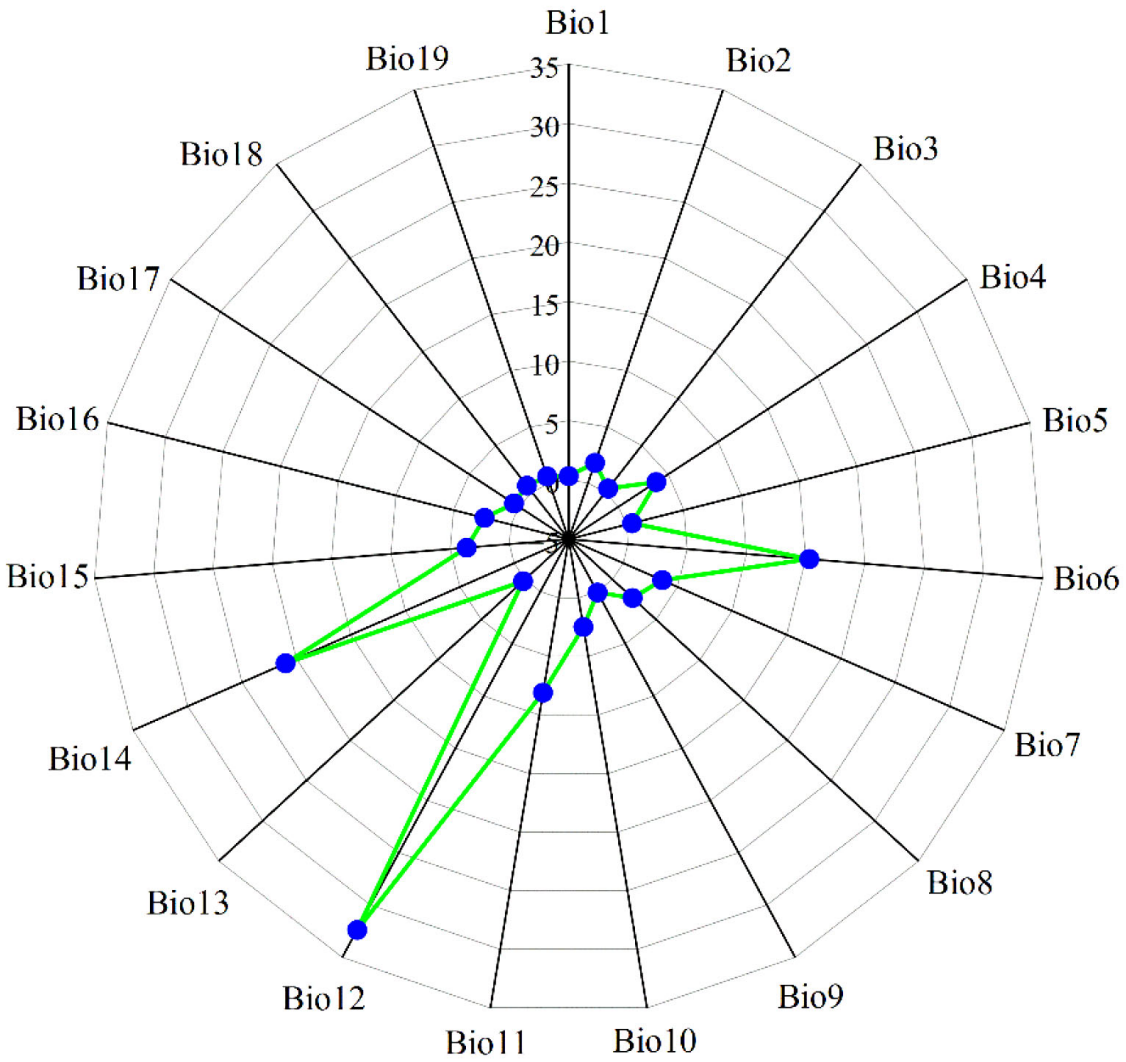
The contribution rate (%) of nineteen bioclimatic variables to *M. officinalis*. Bio1, Annual mean temperature (°C); Bio2, Mean diurnal range (Mean of monthly) (°C); Bio3, Isothermality (Bio2/Bio7) (×100); Bio4, Standard deviation of temperature seasonality; Bio5, Max temperature of warmest month (°C); Bio6, Min temperature of coldest month (°C); Bio7, Temperature annual range (Bio5-Bio6) (°C); Bio8, Mean temperature of wettest quarter (°C); Bio9, Mean temperature of driest quarter (°C); Bio10, Mean temperature of warmest quarter (°C); Bio11, Mean temperature of coldest quarter (mm); Bio12, Annual precipitation (mm); Bio13, Precipitation of wettest month (mm); Bio14, Precipitation of driest month (mm); Bio15, Variation of precipitation seasonality; Bio16, Precipitation of wettest quarter (mm); Bio17, Precipitation of driest quarter (mm); Bio18, Precipitation of warmest quarter (mm); Bio19, Precipitation of coldest quarter (mm).

**Figure 3:**
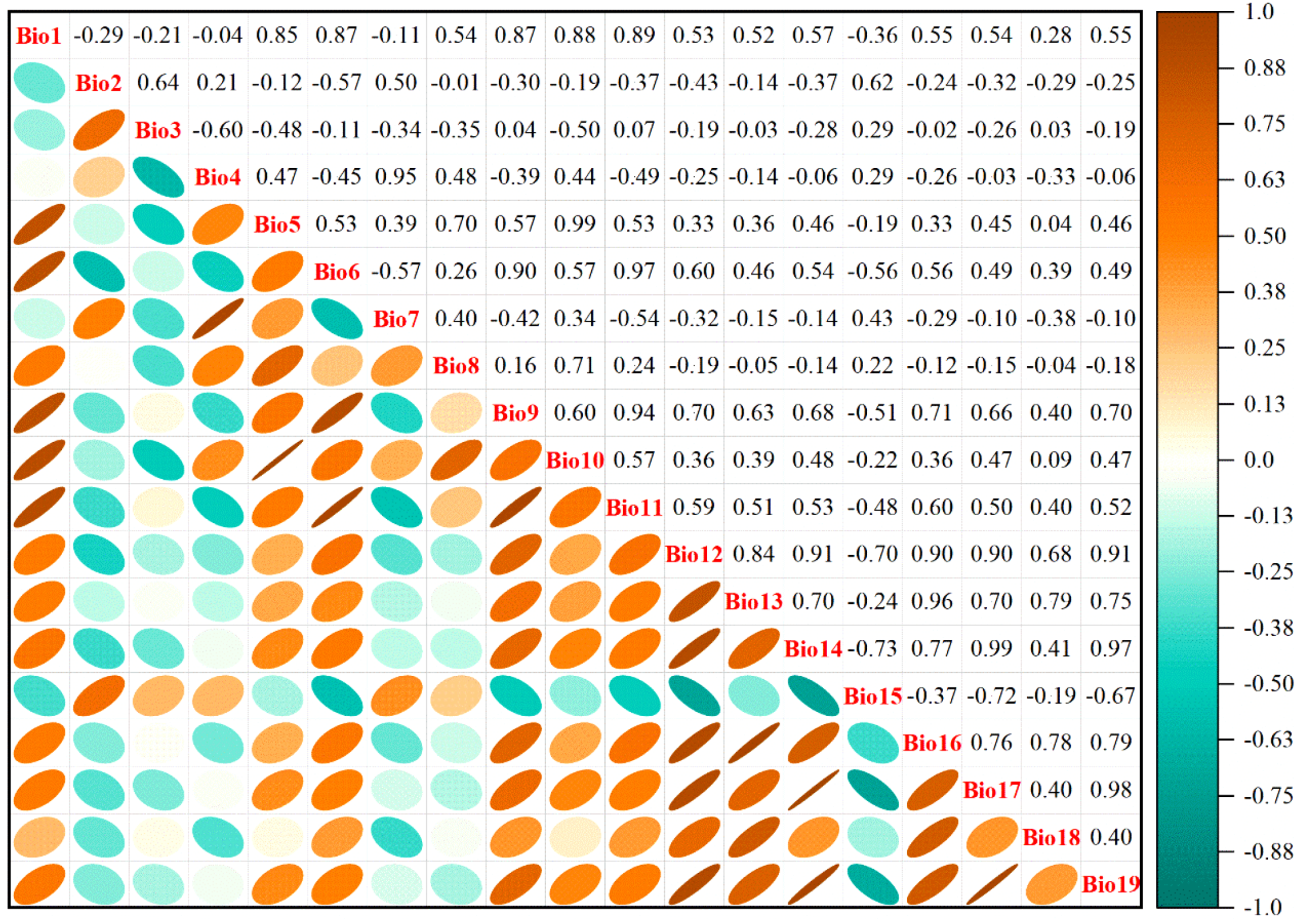
Correlation analysis of nineteen environmental factors with *M. officinalis*.

Multicollinearity among environmental variables, which refers to intercorrelations among two or more predictors in a regression model, can lead to biased model evaluations or impede accurate estimations (Zhang et al., 2024b). The geographical coordinates of 405 *M. officinalis* distribution points in.csv format and 19 Bioclimatic variables under current climate conditions in ASCII format were first imported into MaxEnt 3.4.4 (http://biodiversityinformatics.amnh.org/open_source/maxent/, accessed on 5 September 2024). 75% of the data were randomly selected for model training, while the remaining 25% were used as a test set, which was run 10-repeat cross-validation to determine the contribution rates of the 19 variables to the initial model (**Figure 2**). Subsequently, the attribute values of the 19 bioclimatic variables corresponding to the 405 distribution points were extracted using the “Extract Multi Values to Points” tool in ArcGIS 10.8 software. To address the limitations of analyzing only presence points, we extracted values from both the presence points and a random sample of 1000 background points that represent the environmental gradient across the entire study area. This approach ensures that we consider the full environmental background, following the recommendations of Dormann et al. (2013) and Fourcade et al. (2014). Pearson correlation analysis was conducted on these combined data using IBM SPSS Statistics 26.0 software. Based on the results of the correlation analysis (**Figure 3**), environmental variables with an absolute value of |r| greater than 0.75 and with lower contribution rates in the initial model were eliminated. In addition to Pearson correlation analysis, we applied Variance Inflation Factor (VIF) to assess multicollinearity among predictor variables, ensuring a more robust evaluation. Variables with VIF values exceeding 10 were excluded to guarantee the reliability of our model. Beyond statistical screening (Pearson correlation and VIF), our variable selection incorporated ecological relevance based on established physiological constraints of *Magnoliaceae* species. We retained variables representing: (1) cold stress limitations (Bio6), critical for this subtropical species; (2) seasonal temperature variability (Bio2, Bio4), affecting phenological synchrony; and (3) moisture availability (Bio12, Bio15, Bio18), essential for seedling establishment. This hybrid approach combines statistical rigor with biological realism, addressing known limitations of purely statistical variable selection (Austin, 2002).

### 2.3 Optimization of MaxEnt model

The theoretical basis for our optimization approach follows the principle of balancing model complexity with predictive accuracy (Warren and Seifert, 2011). The regularization multiplier (RM) acts as a smoothing parameter that prevents overfitting by penalizing model complexity. Lower RM values (0.5 in our case) allow for more localized predictions, appropriate for species with narrow ecological niches. The Linear-Quadratic (LQ) feature combination was selected as it captures both linear responses to environmental gradients and potential optimum ranges, consistent with *M. officinalis*’s known ecological requirements (Yang et al., 2020). The ‘ENMeval’ package (R4.2.1) was employed to optimize the MaxEnt model for precisely forecasting the potential distribution range of *M. officinalis* by adjusting two key constraint parameters: the Regularization Multiplier (RM) and the Feature Combination (FC) (Warren and Seifert, 2011). Eight RM parameters, ranging from 0.5 to 4.0 at intervals of 0.5, were established to investigate the model’s performance under different regularization strengths. The MaxEnt model, capable of automatically configuring, was set to encompass five distinct characteristics for the FC settings: Hinge features (H), Linear features (L), Product features (P), Quadratic features (Q), and Threshold features (T). In this study, nine FC parameters were defined: H, HPT, L, LQ, LQH, LQHP, LQHPT, QHP, and QHPT. The Akaike Information Criterion Correction (AICc) was used to assess the model’s fit and complexity, and both the auc.diff.avg and the or.10p.avg were employed to reduce the risk of overfitting. Ultimately, the regularization multiplier and feature combination with a delta.AICc value of zero were chosen to construct the MaxEnt model (Liu et al., 2024a).

### 2.4 Model establishment and evaluation

The 405 data points of *M. officinalis* in.csv format and the selected nine bioclimatic variable data in ASCII format were imported into MaxEnt 3.4.4 software. To validate the model’s accuracy, 75% of the *M. officinalis* sample distribution data were randomly selected for model training, while the remaining 25% were used as a test set. During the optimization process, parameters of RM=0.5 and FC=LQ were set, and the model was run with a maximum of 1000 iterations in the parameters, the calculation was repeated 10 times, and both response curves and jackknife functionality were selected for use. In the MaxEnt modeling process, we used background data points, randomly selected from the study area at a ratio of 3:1 with presence data, ensuring they were distinct from presence points to enhance model accuracy. The output format of the model was set to Logistic mode. The predictive ability of the model was assessed using the Area Under the Curve (AUC) of the Receiver Operating Characteristic (ROC) curve. The AUC value, representing the area under the ROC curve, is an indicator of model accuracy unaffected by the proportion of positive to negative samples. A higher AUC value indicates better model performance in simulating the relationship between the geographical distribution of the target species and environmental factors. The predictive accuracy of the model is graded into five levels: excellent (AUC > 0.9), good (0.8 < AUC ≤ 0.9), fair (0.7 < AUC ≤ 0.8), poor (0.6 < AUC ≤ 0.7), and failed (0.5 < AUC ≤ 0.6) (Araújo et al., 2005).

### 2.5 Classification of the suitable habitat for M. officinalis

The presence probabilities of *M. officinalis*, as predicted by the MaxEnt model (averaged over 10 simulations), were imported into ArcGIS 10.8 software. The model’s output in ASCII format was converted to TIF format using the ‘ArcToolbox → Conversion Tools → To Raster → ASCII to Raster’ function to facilitate spatial analysis and visualization. To accurately delineate the potential suitable habitats for *M. officinalis*, the Jenks natural breaks classification method was employed. Considering the actual distribution characteristics of the species, the study area was divided into four suitability classes based on the presence probability values: non-suitable areas (0-0.1), low suitability areas (0.1-0.3), medium suitability areas (0.3-0.5), and high suitability areas (0.5-1) (Sun et al., 2021).

### 2.6 Spatial distribution pattern changes in suitable habitat of M. officinalis

The average outcomes of the 10 repetitions for *M. officinalis* from MaxEnt 3.4.4 software were employed to delineate suitable habitats where the logistic value was ≥0.1, whereas areas with a logistic value of <0.1 were classified as unsuitable. Subsequently, matrices indicating presence/absence (1, 0) under modern and future climate scenarios were constructed. Within these matrices, a value change from 0 to 1 represents an area of gain, where regions that were previously unsuitable become suitable for *M. officinalis*; a change from 1 to 0 indicates a loss area, where once suitable regions are no longer conducive to the growth of *M. officinalis*; and a change from 1 to 1 signifies a stable area, where suitability is maintained across different time periods. To visually display the changes in the spatial pattern of *M. officinalis* suitable habitats, the matrix values were converted into attribute values and visualized using ArcGIS 10.8 software. The spatial analysis capabilities of this software were employed to map the changes in the spatial pattern of suitable habitats for *M. officinalis*, clearly illustrating the distribution of stable, loss, and gain areas.

### 2.7 Core migration of M. officinalis

The average presence probability results (*M. officinalis*_avg.asc) predicted from the MaxEnt model for the current and future three periods under different climate scenarios were imported into ArcGIS 10.8 software. The “ASCII to Raster” tool was utilized to convert the ASCII files into Tiff format. The Tiff files were then reclassified using the “Classify Raster” tool with a threshold of 0.1, where areas with a species presence probability of ≥0.1 were classified as suitable habitats and those with a probability of <0.1 as unsuitable. The suitable habitat layer was selected, and the “raster to point” tool was applied to convert the raster dataset into point features. Subsequently, the “mean center” tool was used to calculate the centroids of the suitable habitats. To reveal the spatiotemporal evolution of suitable habitats for *M. officinalis* under various Shared Socioeconomic Pathways (SSP) scenarios, the “point merge” tool was employed to aggregate distribution centroid data from different time periods within the same SSP scenario. Finally, the “points to line” tool was used to connect all centroids, visually depicting the migration process of the central areas of suitable habitats for *M. officinalis*.

## 3 Results

### 3.1 Model optimization and accuracy evaluation

Parameter optimization of the MaxEnt model using the ENMeval package indicated that the optimal feature combination was FC = LQ and RM = 0.5, yielding a delta.AICc of zero, whereas the default parameters (FC = LQHPT, RM = 1) resulted in a delta.AICc of 88.6613. Additionally, the refined model exhibited substantially decreased AUC.DIFF and OR10 values compared to the default parameters, with reductions of approximately 31.49% and 59.20%, respectively (**Table 1**).

**Table 1:** Evaluation metrics of MaxEnt model generated by ENMeval.

Parameter Settings	FC	RM	Delta.AICc	AUC.DIFF	OR10
Default	LQHPT	1	88.6613	0.0482	0.3125
Optimized	LQ	0.5	0	0.0330	0.1275

FC, feature combination; RM, regularization multiplier.

**Figure 4:**
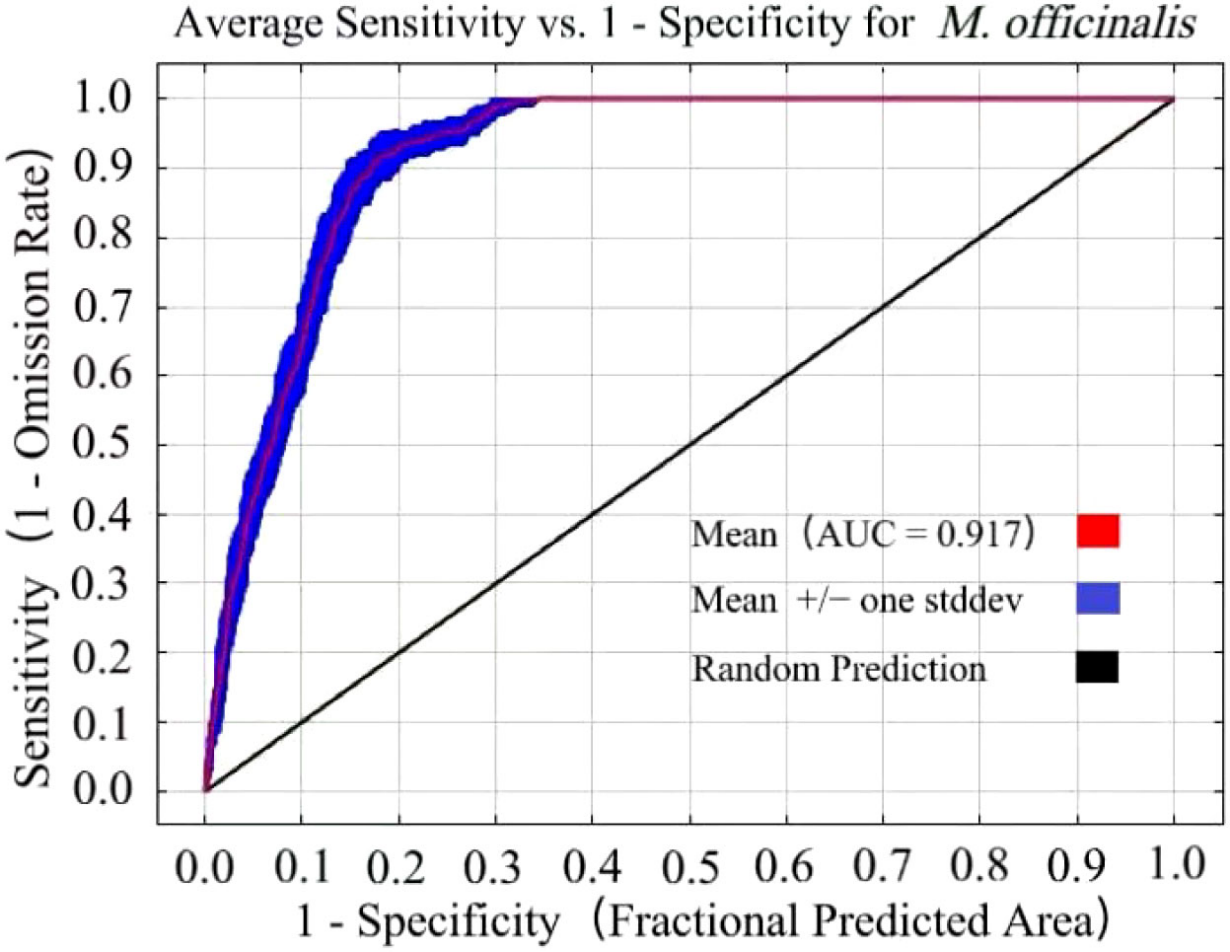
The receiver operating characteristic (ROC) curve derived from the results of ten simulations for *M. officinalis*.

With the optimized parameter settings (RM= 0.5 and FC=LQ), the AUC value achieved was 0.917 (**Figure 4**). Based on AUC evaluation criteria, this score signifies that the model possesses exceptionally high predictive precision. Utilizing these parameters, the potential distribution of *M. officinalis* was forecasted for three future periods under three Shared Socioeconomic Pathways (SSP) scenarios, yielding AUC values consistently at 0.90. Therefore, it is ascertained that the model reliably predicts potential distribution areas for *M. officinalis* across China.

### 3.2 Primary environmental variables

**Figure 5:**
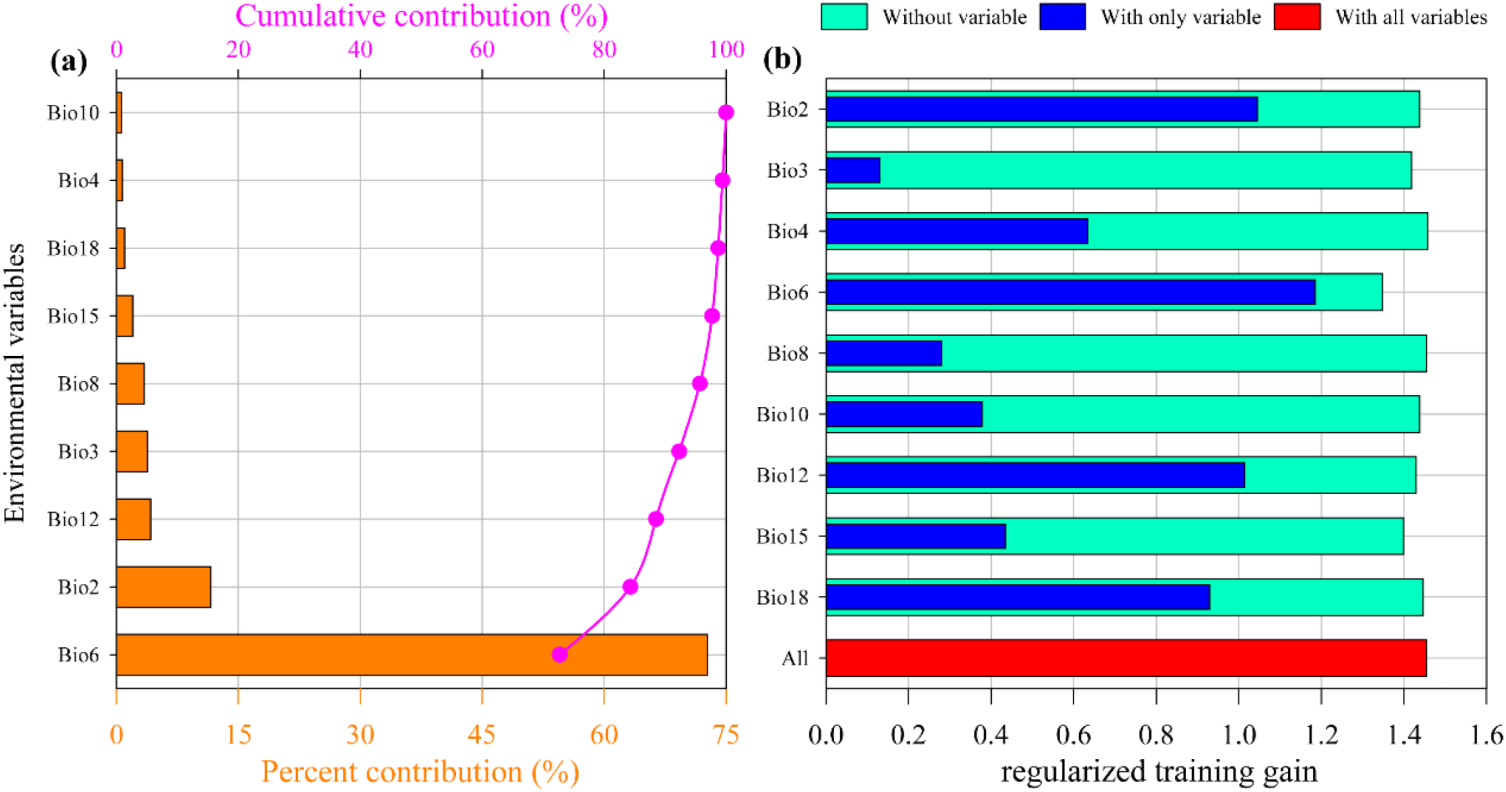
The contribution rate **(a)** and Jackknife test **(b)** result of environmental factors for *M. officinalis*.

The contribution rates of environmental factors (**Figure 5a**) indicate that the most significant factor affecting the suitable distribution of *M. officinalis* is the Minimum temperature of the coldest month (Bio6), with a contribution rate of 72.7%. The factors with the second and third highest contribution rates are the Mean diurnal range (Mean of monthly) (Bio2) and Annual precipitation (Bio12), with contribution rates of 11.6% and 4.2%, respectively. In contrast, the factor with the smallest contribution rate is the Mean temperature of the warmest quarter (Bio10), at only 0.6%. The cumulative contribution rate of the top three environmental factors is 88.5%, which suggests that they dominate the suitable distribution of *M. officinalis*. Through the analysis of the regularized training gain of the jackknife test for the impact of nine key environmental factors on the distribution of *M. officinalis*, it is known that the Minimum temperature of the coldest month (Bio6), Mean diurnal range (Mean of monthly) (Bio2), and Annual precipitation (Bio12) have a significant impact on the distribution of *M. officinalis* (**Figure 5b**). By integrating the results of model contribution rates and jackknife tests, we have unveiled the key environmental factors affecting the suitable distribution of *M. officinalis*: Minimum temperature of the coldest month (Bio6), Mean diurnal range (Mean of monthly) (Bio2), and Annual precipitation (Bio12).

**Figure 6:**
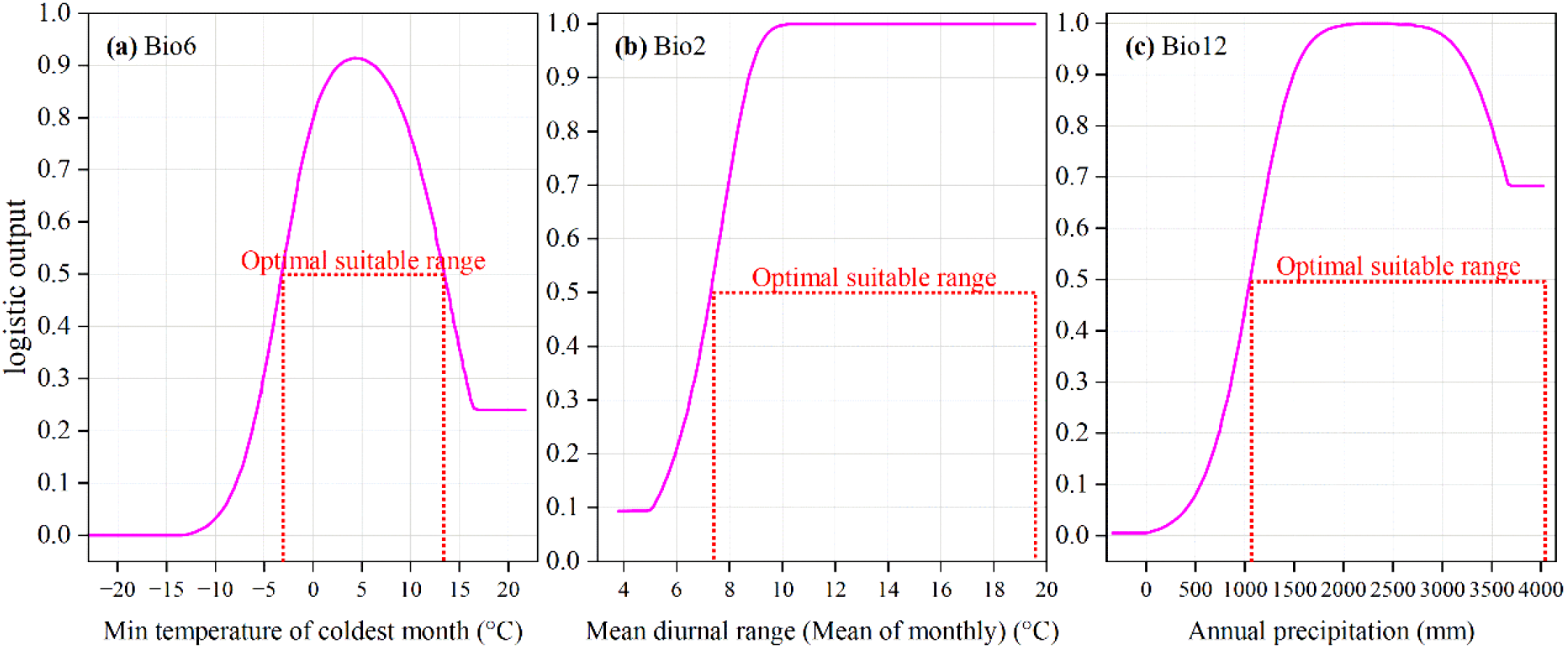
Response curves of *M. officinalis* to three key environmental factors. **(a)** Min temperature of coldest month (°C); **(b)** Mean diurnal range (Mean of monthly) (°C); **(c)** Annual precipitation (mm).

It is generally understood that environmental factor values are considered suitable for a species’ growth when its presence probability is greater than 0.5. From the response curves of the three dominant environmental factors (**Figure 6**), it can be observed that the presence probability of *M. officinalis* first increases and then decreases with the increase in the minimum temperature of the coldest month and annual precipitation. Conversely, as the mean diurnal range gradually increases, the presence probability of *M. officinalis* tends to rise gradually and maintain a peak value. Additionally, *M. officinalis* is more suitable for growth when the minimum temperature of the coldest month is between -3.08 to 13.33°C, with the presence probability peaking at 4.11°C. The presence probability of *M. officinalis* is higher when the annual precipitation is between 1067.82 to 4067.96 mm, peaking at 2000 to 2694.92 mm. The suitable range for the mean diurnal range for the growth of *M. officinalis* is 7.40 to 19.62°C, and its presence probability remains at a peak when the mean diurnal range is between 10.17 to 19.62°C.

### 3.3 Suitable habitats for M. officinalis under current climate scenarios

**Figure 7:**
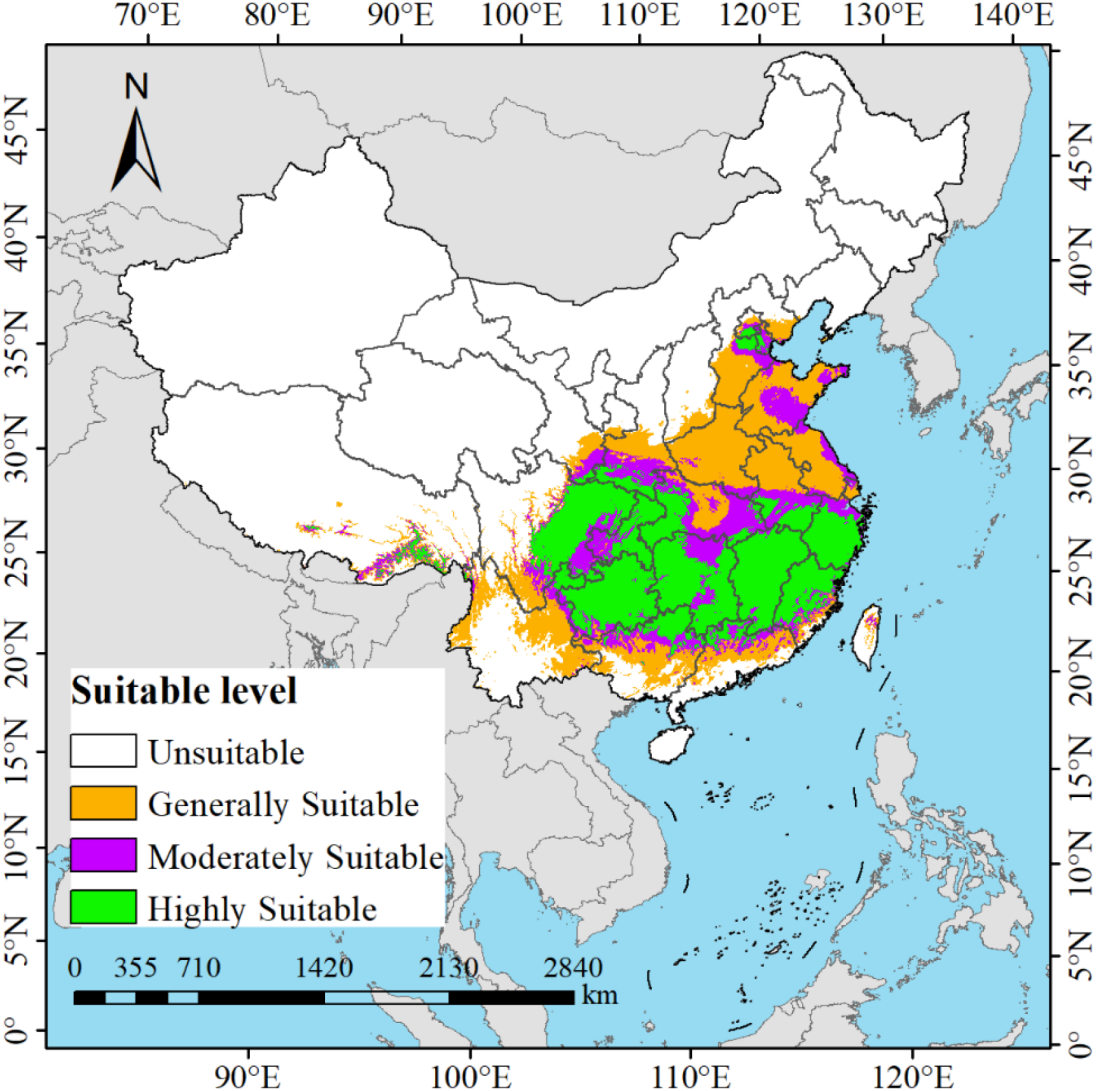
Potential distribution of *M. officinalis* under different current climate scenarios in China.

Based on the current environmental factors and distribution point data of *M. officinalis*, the prediction results of the MaxEnt model show that the suitable habitat of *M. officinalis* under current climate conditions is mainly concentrated in the southern regions of China (**Figure 7**). Under the current climate conditions, the total suitable habitat area of *M. officinalis* in China reaches 254.70×10^4^ km^2^, accounting for about 26.53% of the country’s land area. Among this, the high suitability area, which accounts for approximately 103.65×10^4^ km^2^ or 10.80% of the national land area, is primarily located in most parts of Guizhou Province, Chongqing Municipality, Fujian Province, Zhejiang Province, Jiangxi Province, as well as in the western Hubei and Hunan provinces, the central and eastern Sichuan Province, the northern Guangdong Province, the northern Guangxi Zhuang Autonomous Region, the southern Anhui Province, and the junction of Beijing Municipality, Tianjin Municipality, and Hebei Province. The medium suitability area covers about 52.76×10^4^ km^2^, representing 5.50% of China’s land area, and mainly includes the central and northern Hunan Province, the southwestern Guizhou Province, the eastern Sichuan Province, the western Chongqing Municipality, the southern Shaanxi Province, the southern Gansu Province, the eastern Hubei Province, the southern Anhui Province, the central Jiangxi Province, and the central Zhejiang Province. The low suitability area, with an area of 98.29×10^4^ km^2^, accounts for 10.24% of the national land area, and is widely distributed across most regions of Jiangsu Province, Anhui Province, Shandong Province, Henan Province, Hubei Province, Yunnan Province, as well as in the southern Sichuan Province, the southern Tibet Autonomous Region, the southern Gansu Province, the southern Shaanxi Province, the central Guangdong Province, the central Guangxi Zhuang Autonomous Region, the central Taiwan Province, the northern Zhejiang Province, the central and eastern Hebei Province, and the northern Beijing Municipality.

### 3.4 Potential suitable distribution areas of M. officinalis under future climate conditions

**Figure 8:**
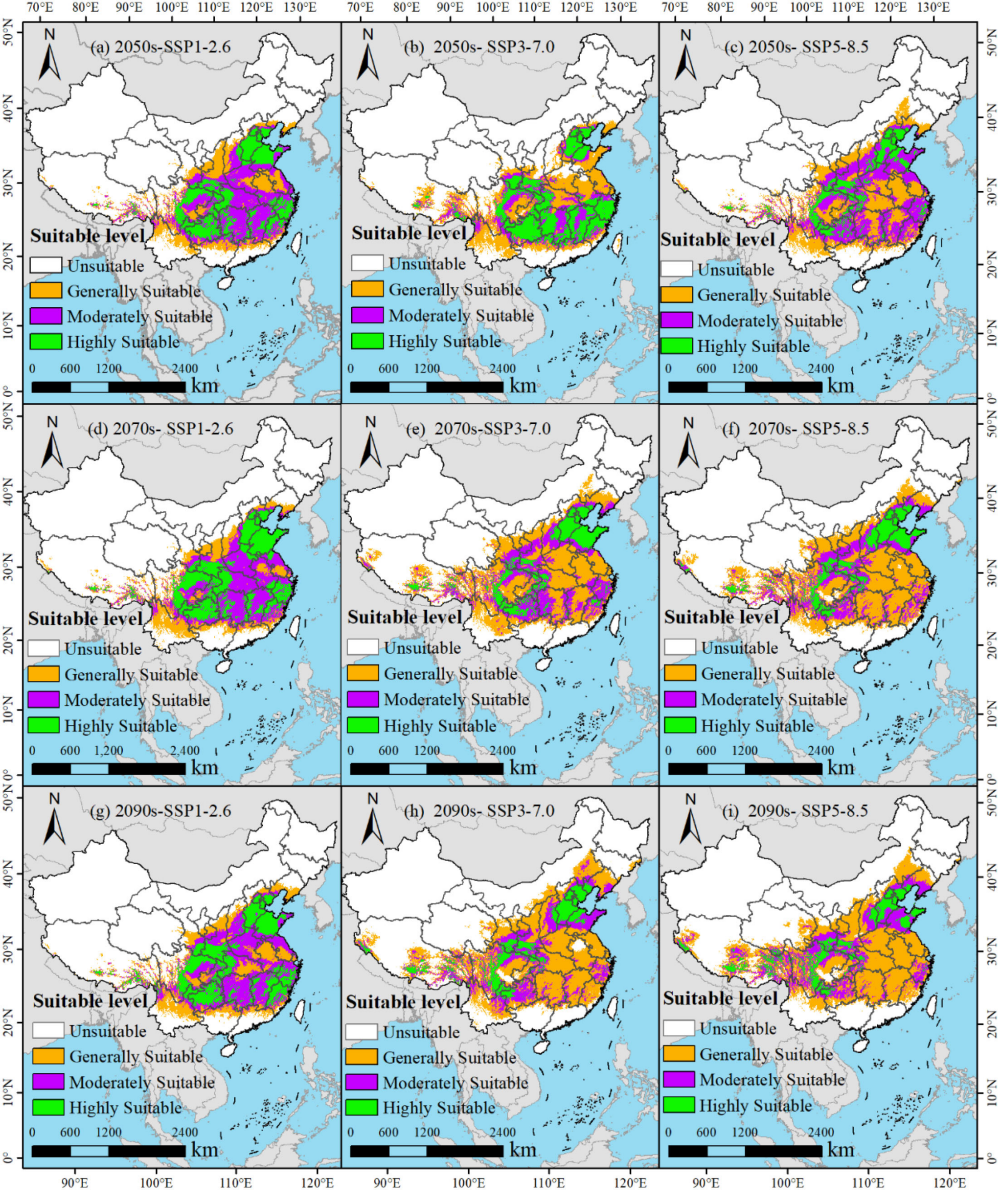
**(a-i)** Potential distribution of *M. officinalis* in China under future climate change scenarios.

**Figure 9:**
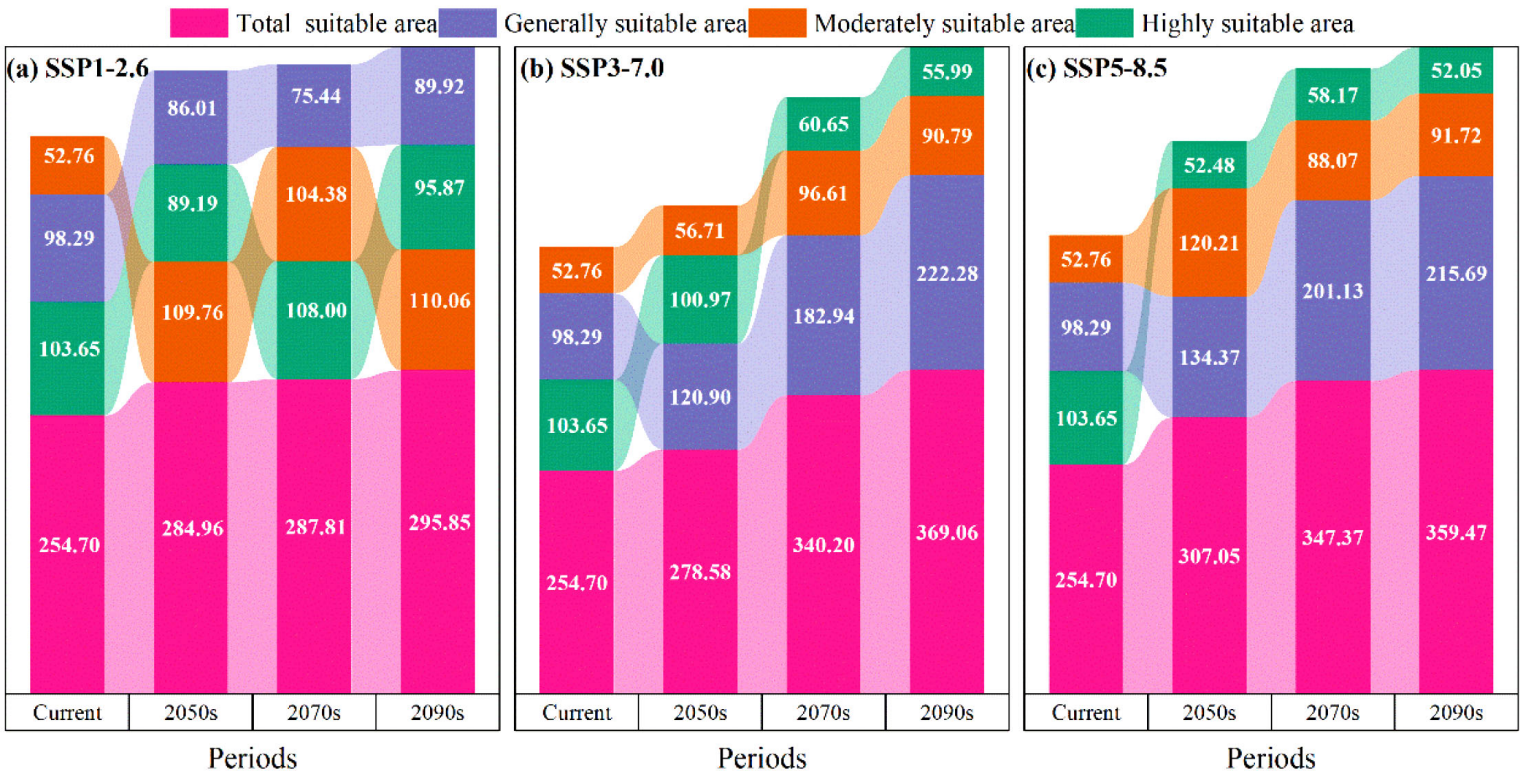
**(a-c)** Predicted suitable area for the *M. officinalis* for the current, 2050s, 2070s, and 2090s under different climate scenarios (unit: 10^4^ km^2^).

Under the SSP1-2.6, SSP3-7.0, and SSP5-8.5 climate change scenarios, the potential suitable distributions of *M. officinalis* for the 2050s, 2070s, and 2090s are forecasted using the optimized MaxEnt model (**Figure 8**). The results indicate that from the present to the 2050s under the SSP1-2.6 scenario, the total suitable habitat for *M. officinalis* increased by 30.26×10^4^ km^2^ (**Figure 9a**). This increase is primarily due to a significant expansion of the moderately suitable habitat by 57.00×10^4^ km^2^, while the low and high suitability areas decreased by 12.28×10^4^ km^2^ and 14.46×10^4^ km^2^, respectively. By the 2070s, compared to the 2050s, the total suitable habitat only increased by 2.86×10^4^ km^2^, with a notable increase in the high suitability area by 18.81×10^4^ km^2^, and reductions in the low and moderate suitability areas by 10.57×10^4^ km^2^ and 5.38×10^4^ km^2^, respectively. By the 2090s, the total suitable habitat increased by 8.04×10^4^ km^2^ compared to the 2070s, with the low and moderate suitability areas showing increases of 14.49×10^4^ km^2^ and 5.68×10^4^ km^2^, respectively, and a decrease in the high suitability area by 12.12×10^4^ km^2^.

From the present to the 2050s under the SSP3-7.0 climate scenario (**Figure 9b**), the total suitable habitat for *M. officinalis* increased by 23.88×10^4^ km^2^. This increase was characterized by expansions in the low and high suitability areas by 22.61×10^4^ km^2^ and 3.95×10^4^ km^2^, respectively, while the moderately suitable area decreased by 2.68×10^4^ km^2^. By the 2070s, compared to the 2050s, the total suitable habitat for *M. officinalis* increased by 61.61×10^4^ km^2^, with significant expansions in the low and moderately suitable areas by 62.03×10^4^ km^2^ and 39.90×10^4^ km^2^, respectively, and a reduction in the high suitability area by 40.32×10^4^ km^2^. By the 2090s, the total suitable habitat increased by an additional 28.86×10^4^ km^2^ compared to the 2070s, with a notable expansion in the low suitability area by 39.34×10^4^ km^2^, and reductions in the moderately and high suitability areas by 5.82×10^4^ km^2^ and 4.67×10^4^ km^2^, respectively.

From the present to the 2050s under the SSP5-8.5 climate scenario, the total suitable habitat for *M. officinalis* expanded by 52.36×10^4^ km^2^ (**Figure 9c**). This expansion mainly resulted from the increases in the low and high suitability areas by 36.08×10^4^ km^2^ and 67.44×10^4^ km^2^, respectively, while the moderately suitable area decreased by 51.17×10^4^ km^2^. By the 2070s, compared to the 2050s, the total suitable habitat for *M. officinalis* further increased by 40.32×10^4^ km^2^, with significant growth in the low and high suitability areas by 66.77×10^4^ km^2^ and 5.68×10^4^ km^2^, respectively, and a reduction in the moderately suitable area by 32.14×10^4^ km^2^. By the 2090s, the total suitable habitat increased by an additional 12.10×10^4^ km^2^ compared to the 2070s, with the low and moderately suitable areas showing increases by 14.56×10^4^ km^2^ and 3.65×10^4^ km^2^, respectively, and a decrease in the high suitability area by 6.11×10^4^ km^2^.

The optimized MaxEnt model was employed to forecast potential suitable distributions of *M. officinalis* under SSP1-2.6, SSP3-7.0, and SSP5-8.5 climate scenarios for the 2050s, 2070s, and 2090s (**Figure 8**). Analysis of habitat suitability projections revealed distinct temporal patterns across the three scenarios (**Table 1**).

Under all scenarios, the total suitable habitat area for *M. officinalis* is projected to increase from the present to the 2090s, though with differing magnitudes and distributional patterns. The SSP5-8.5 scenario shows the most substantial expansion in total suitable habitat (104.78×10^4^ km^2^), followed by SSP3-7.0 (114.35×10^4^ km^2^) and SSP1-2.6 (41.16×10^4^ km^2^). However, a notable finding is that despite overall habitat expansion, highly suitable areas exhibit vulnerability under more severe climate change scenarios.

Temporal analysis reveals three key patterns. First, a habitat suitability shift gradient is evident, with moderately suitable habitats expanding most significantly under SSP1-2.6 (57.00×10^4^ km^2^), while low suitability areas predominate expansion under SSP3-7.0 and SSP5-8.5. Second, the rate of change accelerates in the 2070s under SSP3-7.0 and SSP5-8.5 scenarios but stabilizes under SSP1-2.6. Third, highly suitable habitats show greatest sensitivity to severe climate scenarios, with substantial reductions under SSP3-7.0 (40.32×10^4^ km^2^) and SSP5-8.5 during the 2070s.

Geographically, habitat expansion primarily occurs in northwestern regions under all scenarios, with variation in the extent and quality of new suitable areas. The most significant contractions of high-quality habitat occur in southeastern regions, particularly in parts of Fujian, Zhejiang, and Jiangxi provinces.

### 3.5 Spatial changes of potential suitable areas of M. officinalis under different climate scenarios in the future

**Figure 10:**
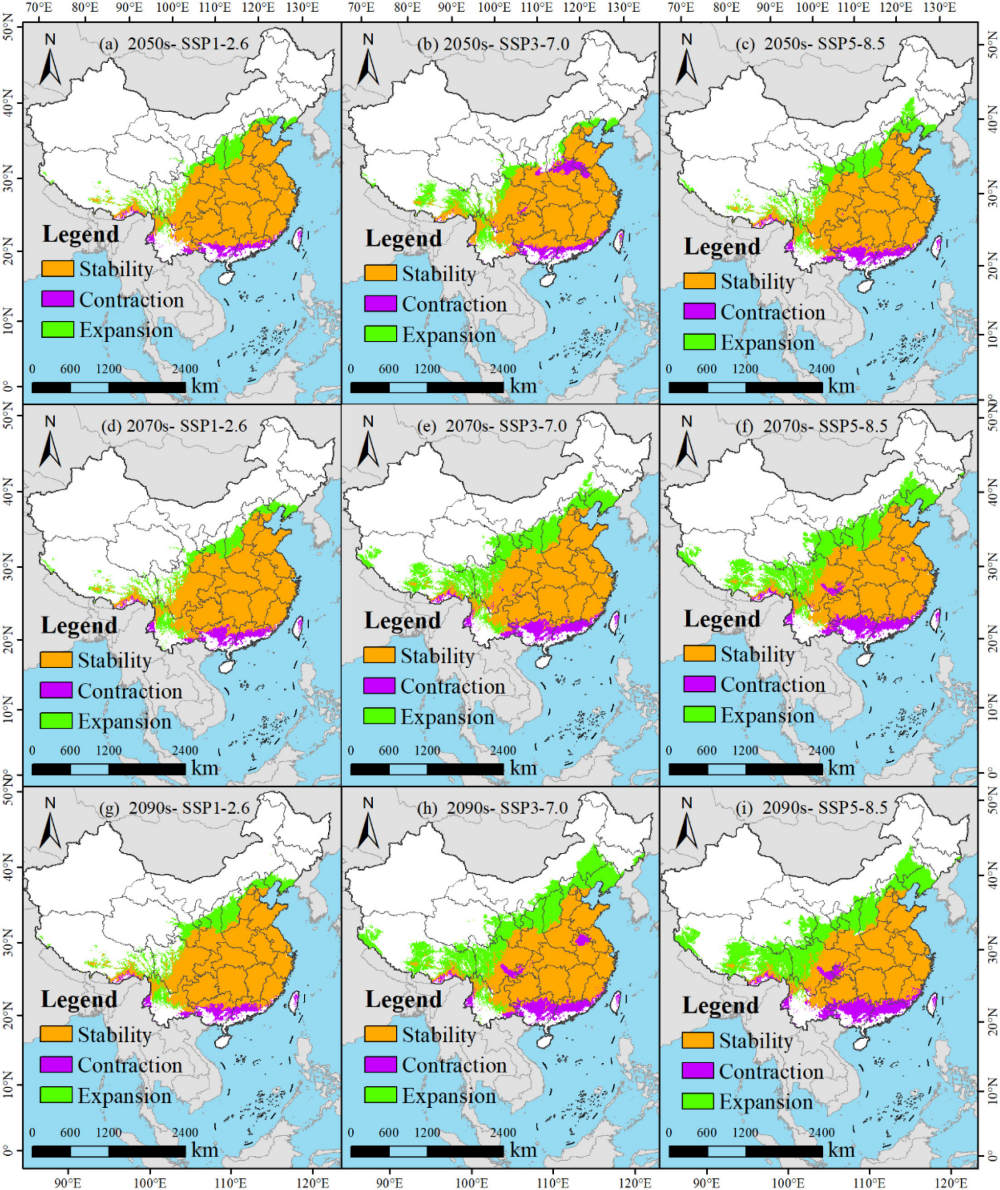
**(a-i)** Dynamic change of the predicted potentially suitable areas for *M. officinalis* (compared to the current range).

Based on the spatial analysis capabilities of ArcGIS software, a comparative analysis can be conducted between the potential suitable habitats of *M. officinalis* in the future under the same climate scenario and the current suitable habitats. This analysis reveals the distribution of retained, lost, and gained areas of suitable habitats for *M. officinalis* in the future (**Figure 10**; **Table 2**), which is crucial for conservation and adaptation planning.

**Table 2:** Spatial dynamics of suitable habitats for *M. officinalis* in response to diverse future climate scenarios (compared to the current range).

Period	Area (10^4^ km^2^)			Rate of change (%)		
	Stability	Contraction	Expansion	Stability	Contraction	Expansion
2050s-SSP1-2.6	295.19	19.29	56.72	79.52	5.20	15.28
2070s-SSP1-2.6	291.93	22.57	63.65	77.20	5.97	16.83
2090s-SSP1-2.6	293.74	20.76	71.76	76.05	5.37	18.58
2050s-SSP3-7.0	283.64	30.87	60.41	75.65	8.23	16.11
2070s-SSP3-7.0	282.15	32.33	138.25	62.32	7.14	30.54
2090s-SSP3-7.0	274.69	39.78	181.31	55.41	8.02	36.57
2050s-SSP5-8.5	288.95	25.52	90.27	71.39	6.31	22.30
2070s-SSP5-8.5	274.72	39.76	154.68	58.56	8.47	32.97
2090s-SSP5-8.5	266.17	48.30	177.82	54.07	9.81	36.12

Specifically, under the SSP1-2.6 climate scenario, in the 2050s, the retained area of the suitable habitat for *M. officinalis* is 295.19×10^4^ km^2^, accounting for 79.52% of the total area; the lost area is 19.29×10^4^ km^2^, with a loss rate of 5.2%; and the gained area is 56.72×10^4^ km^2^, with an increase rate of 15.28% (**Figure 10a**; **Table 2**). By the 2070s, the retained area of the suitable habitat slightly decreases to 291.93×10^4^ km^2^, with a retention rate of 77.2%; the lost area increases to 22.57×10^4^ km^2^, with a loss rate of 5.97%; and the gained area is 63.65×10^4^ km^2^, with an increase rate of 16.83% (**Figure 10d**; **Table 2**). By the 2090s, the retained area of the suitable habitat further decreases to 293.74×10^4^ km^2^, with a retention rate of 76.05%; the lost area is 20.76×10^4^ km^2^, with a slightly decreased loss rate of 5.37%; and the gained area expands to 71.76×10^4^ km^2^, with an increase rate of 18.58% (**Figure 10g**; **Table 2**).

Under the SSP3-7.0 climate scenario for the 2050s, the area of suitable habitat for *M. officinalis* that is expected to be retained is 283.64×10^4^ km^2^, which is 75.65% of the total suitable area; meanwhile, an area of 30.87×10^4^ km^2^ is projected to be lost, accounting for 8.23% of the total suitable area; and the area of suitable habitat that is anticipated to be gained is 60.41×10^4^ km^2^, making up 16.11% of the total suitable area (**Figure 10b**; **Table 2**). By the 2070s, the retained area of the suitable habitat for *M. officinalis* is forecasted to slightly decrease to 282.15×10^4^ km^2^, with a retention rate of approximately 62.32%; the lost area is expected to increase to 32.33×10^4^ km^2^, with a loss rate of 7.14%; and the gained area is projected to significantly increase to 138.25×10^4^ km^2^, with an increase rate of 30.54% (**Figure 10e**; **Table 2**). Entering the 2090s, the retained area of the suitable habitat for *M. officinalis* is anticipated to further reduce to 274.69×10^4^ km^2^, with a retention rate of 55.41%; the lost area is expected to rise to 39.78×10^4^ km^2^, with a loss rate of 8.02%; and the gained area is forecasted to expand to 181.31×10^4^ km^2^, with an increase rate of 36.57% (**Figure 10h**; **Table 2**).

Under the SSP5-8.5 climate scenario, the suitable growing area for *M. officinalis* in the 2050s is anticipated to be maintained at 288.95×10^4^ km^2^, which is 71.39% of the total suitable area. In the meantime, an area of 25.52×10^4^ km^2^ is expected to no longer be suitable for the growth of *M. officinalis*, making up 6.31% of the total; and the newly added suitable area is projected to be 90.27×10^4^ km^2^, constituting 22.3% of the total suitable area (**Figure 10c**; **Table 2**). By the 2070s, the retained area of the suitable growing region is expected to contract to 274.72×10^4^ km^2^, with a retention rate of 58.56%; the area lost is anticipated to increase to 39.76×10^4^ km^2^, with a loss rate of 8.47%; and the increased suitable area is expected to expand to 154.68×10^4^ km^2^, with an increase rate of 32.97% (**Figure 10f**; **Table 2**). Moving further into the 2090s, the retained area of the suitable growing region is forecasted to decrease to 266.17×10^4^ km^2^, with a retention rate of 54.07%; the lost area is projected to reach 48.3×10^4^ km^2^, with a loss rate of 9.81%; and the increased suitable area is anticipated to further rise to 177.82×10^4^ km^2^, with an increase rate of 36.12% (**Figure 10i**; **Table 2**). These data outline the changing trends of the suitable growing areas for *M. officinalis* over time under the SSP5-8.5 climate scenario.

The spatial persistence, contraction, and expansion of suitable habitats for *M. officinalis* were analyzed across future climate scenarios (**Figure 10**, **Table 2**). This analysis identified three distinct spatial patterns with important conservation implications. First, habitat stability shows a consistent declining trend across all scenarios from the 2050s to the 2090s. The most stable pattern occurs under SSP1-2.6, with 79.52% habitat retention in the 2050s declining gradually to 76.05% by the 2090s. In contrast, SSP5-8.5 and SSP3-7.0 show more dramatic reductions in habitat stability, declining to 54.07% and 55.41% respectively by the 2090s. Second, the spatial pattern of habitat loss reveals important ecological vulnerabilities. Under all scenarios, habitat loss particularly affects southeastern regions, with core areas in Fujian, Zhejiang, and southern Anhui provinces. Loss rates increase with scenario severity, reaching 9.81% under SSP5-8.5 by the 2090s compared to 5.37% under SSP1-2.6. Third, habitat expansion exhibits a northwestern directional shift across all scenarios, with expansion rates increasing with scenario severity. By the 2090s, expansion rates reach 18.58%, 36.57%, and 36.12% under SSP1-2.6, SSP3-7.0, and SSP5-8.5 respectively. This expansion predominantly occurs in previously marginal areas of Shaanxi, Sichuan, and western Hubei provinces.

These spatial dynamics suggest a climate-driven northwestward migration of suitable habitats for *M. officinalis*, with more severe scenarios accelerating this directional shift. While total suitable area increases under all scenarios, the spatial reconfiguration indicates potential ecological disruption, with significant implications for conservation planning and assisted migration strategies.

### 3.6 Centroid migration dynamics within the suitable habitats of *M. officinalis*

**Figure 11:**
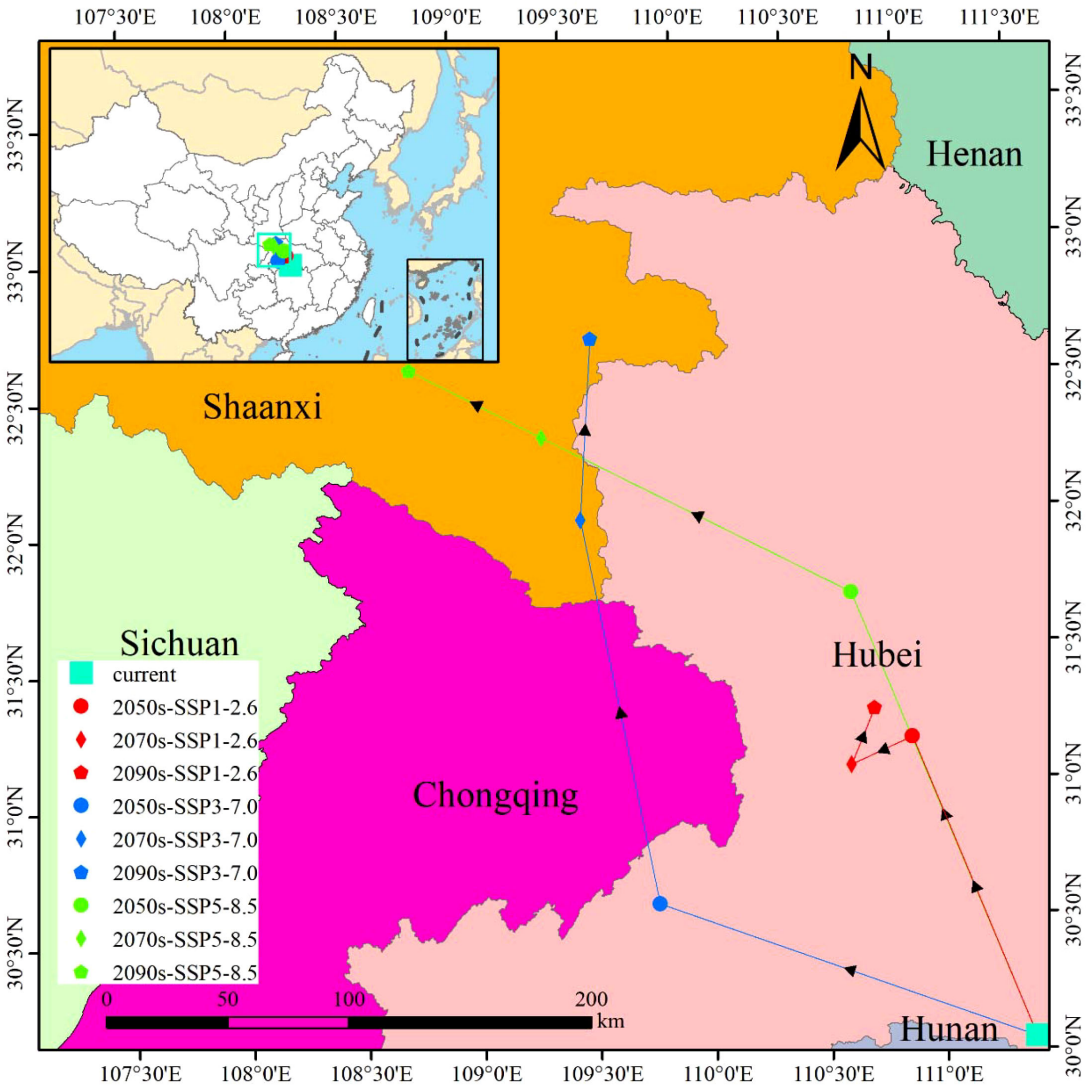
The shift trend of centroid distribution of *M. officinalis.*.

Under current climatic conditions, the centroid of the suitable habitat for *M. officinalis* is in Xijia Ping Town, Songzi County, Hubei Province, China (111°23′E, 30°30′N). According to predictions under different Shared Socioeconomic Pathway (SSP) climate scenarios, significant changes are expected in the habitat center for *M. officinalis* over the coming decades (**Figure 11**).

Under the SSP1-2.6 climate scenario for the 2050s, the habitat centroid is projected to shift northwest by 132.67 km to Gao Lan Town, Xingshan County, Hubei Province (110°55′E, 31°10′N). By the 2070s, it is expected to move a further 27.87 km to the southwest, settling in Shuitianba Town, Zigui County (110°39′E, 31°05′N), and by the 2090s, it is anticipated to move 25.13 km to the northeast, finally positioning in Zhaojun Town, Xingshan County (110°46′E, 31°17′N).

Under the SSP3-7.0 scenario for the 2050s, the centroid is expected to shift northwest by 166.01 km to Hua Ping Town, Jianshi County, Hubei Province (109°47′E, 30°36′N). By the 2070s, it is projected to move an additional 159.90 km northwest to Baijia Town, Zhenping County, Shaanxi Province (109°31′E, 32°01′N), and by the 2090s, it is expected to migrate 74.15 km to the northeast, ultimately locating in Jinzhai Town, Xunyang County, Shaanxi Province (109°35′E, 32°41′N).

Under the SSP5-8.5 scenario for the 2050s, the habitat centroid is anticipated to shift northwest by 196.86 km to Songluo Town, Shennongjia Forestry District, Hubei Province (110°41′E, 31°43′N). By the 2070s, it is expected to move a further 143.57 km northwest to Chang’an Town, Pingli County, Shaanxi Province (109°21′E, 32°20′N), and by the 2090s, it is projected to migrate 61.84 km to the northwest, finally settling in Liushui Town, Hanbin District, Shaanxi Province (108°46′E, 32°36′N).

## 4 Discussion

### 4.1 Optimization and assessment of maxent model for *M. officinalis*

The MaxEnt model is widely used in ecology and biogeography due to its low data requirements, high predictive accuracy, and ease of operation (Yackulic et al., 2013). However, the default parameter settings of the model may not be suitable for all species and regions, especially when simulating the potential distribution of species, where model complexity significantly affects predictive accuracy and the model’s transferability. Optimizing parameters, such as adjusting the regularization multiplier and selecting appropriate feature types, is crucial for improving the model’s predictive performance and generalization ability (Lissovsky and Dudov, 2021). Through optimization, the model can more reasonably reflect the species’ response to environmental factors, reduce the risk of overfitting, and enhance the accuracy of predictions in new areas (Zhao et al., 2022). Therefore, optimizing the MaxEnt model not only improves predictive precision but also enhances the model’s applicability and flexibility in different environmental and climatic contexts (Zhao et al., 2024), which has significant academic and practical significance for understanding and predicting the impact of climate change on species distribution.

The MaxEnt model’s predictive accuracy and generalization ability were significantly enhanced through parameter optimization using the ENMeval package in this study. The optimized model (FC = LQ and RM = 0.5) achieved a delta.AICc of zero, indicating that the model is optimal in terms of information criteria, as it represents the best balance between model complexity and data fitting. Similar to existing species distribution models like *Actinidia chinensis* (Wang et al., 2024b), *Liriodendron chinense* (Bai et al., 2024), and *Cunninghamia lanceolata* (Zhao et al., 2021), our study confirms MaxEnt’s effectiveness. Despite differing ecological contexts, these models share comparable predictive accuracy, underscoring MaxEnt’s reliability for projecting species’ distributions. In contrast, the model using default parameters (FC = LQHPT, RM = 1) resulted in a delta.AICc of 88.6613, suggesting that the model complexity under default parameters is too high, potentially leading to overfitting. Additionally, the optimized model showed significant improvements in performance metrics AUC.DIFF and OR10, with reductions of approximately 31.49% and 59.20%, respectively. The decrease in AUC.DIFF implies an enhanced ability of the model to distinguish between areas of species distribution and non-distribution, while the reduction in OR10 indicates a decrease in uncertainty when predicting species distribution. These improvements are crucial for increasing the model’s predictive accuracy in new areas, especially in studies on the impact of climate change on species distribution, providing more reliable predictive results for ecological conservation and species management.

An AUC value of 0.917 was obtained for the prediction of *M. officinalis* using the optimized MaxEnt model in this study, indicating that the model possesses extremely high predictive accuracy, which is consistent with previous studies that have recognized the high accuracy of MaxEnt models in predicting species distributions (Zhan et al., 2022; Shi et al., 2023). Under the AUC evaluation criterion, a score of 0.917 signifies that the model has a very strong ability to distinguish between areas of species distribution and non-distribution. With these optimized parameters, the potential distribution of *M. officinalis* under three different time periods and three Shared Socioeconomic Pathways (SSP) scenarios was predicted, with AUC values consistently around 0.90. This result not only confirms the high predictive accuracy of the model but also demonstrates its stability and reliability under different environmental and socioeconomic scenarios, aligning with the findings of other studies that have shown the robustness of MaxEnt models across various conditions (Gao et al., 2024; Meena et al., 2024). Such findings are of significant academic value for understanding and predicting the impact of climate change on species distribution and provide a scientific basis for the development of conservation strategies and adaptation measures. However, it is important to note that MaxEnt models can sometimes be sensitive to sampling bias and may overfit the data, which can affect their transferability and predictive accuracy in new areas or under different conditions. Therefore, while the results are promising, they should be interpreted with caution and further validated with independent data.

Our study advances SDM applications for endangered medicinal plants by demonstrating that systematic parameter optimization can improve predictive accuracy by 31.49% (AUC.DIFF reduction) compared to default settings. This methodological refinement is particularly crucial for conservation planning, where model uncertainty directly impacts resource allocation decisions.

### 4.2 Key environmental factors regulating suitable habit of M. Officinalis

The parameters of the MaxEnt model were optimized, and the Jackknife test was conducted, through which the most significant environmental factor affecting the distribution of *M. officinalis* was identified as the minimum temperature of the coldest month (Bio6), with a contribution rate as high as 72.7%. This finding emphasizes the key role of temperature in determining plant distribution, especially under the backdrop of climate change. Following this, the average diurnal temperature range (Bio2) and annual precipitation (Bio12) were also identified as important factors, contributing 11.6% and 4.2%, respectively.

The dominance of minimum temperature of the coldest month (Bio6) as a predictor (72.7% contribution) aligns with *M. officinalis*’s physiological constraints. This species requires a vernalization period for flower bud differentiation, with optimal chilling requirements between 0-7°C for 30-45 days (Yang et al., 2015). Temperatures below -15°C can cause xylem embolism and tissue damage, while insufficient chilling (<0°C for less than 20 days) results in poor flowering and reduced seed set. The identified threshold range (-3.08 to 13.33°C) corresponds closely to these physiological limits. Annual precipitation’s contribution (4.2%) reflects the species’ drought tolerance mechanisms, including deep taproot systems and thick, waxy leaves that reduce water loss. However, the optimal range (1067-4067 mm) suggests that while drought-tolerant, *M. officinalis* benefits from consistent moisture availability during the growing season, particularly for seedling establishment.

Similar studies have also found that the minimum temperature of the coldest month (Bio6) is a key environmental factor regulating the potential suitable distribution for *Angelica dahurica, Prunus avium, Cinnamomum mairei*, and *Cunninghamia lanceolata* in China (Zhou et al., 2021; Qi et al., 2022; Li et al., 2024; Zhang et al., 2024a). This study revealed that among the nine climate and environmental variables, the impact of temperature on the distribution of *M. officinalis* far exceeds that of precipitation. This phenomenon may be caused by multiple factors: firstly, temperature is a core environmental factor for plant growth and development, directly related to the physiological needs and metabolic activities of plants, such as photosynthesis, respiration, and transpiration (Dusenge et al., 2019; Wang and Wang, 2023). Secondly, seasonal temperature fluctuations and extreme events significantly affect plant distribution and diversity (Lloret et al., 2012); a rise in temperature may expand the ecological niche of some species while compressing the living space of others (Foster, 2001). On a broader scale, climatic factors, especially temperature changes, play a decisive role in influencing plant distribution. These findings indicate that temperature not only directly affects plant physiological activities but also indirectly influences plant distribution by regulating ecological niches, competitive relationships, and genetic diversity (Huang et al., 2021). Therefore, in the context of climate change’s impact on plant distribution, temperature typically plays a more critical role than precipitation.

### 4.3 Suitable distribution of M. officinalis induced by climate change

Our study found that under current climatic conditions, *M. officinalis* is primarily distributed in regions such as Guizhou Province, Chongqing Municipality, Hunan Province, Jiangxi Province, Fujian Province, Zhejiang Province, eastern Sichuan Province, northern Guangxi Zhuang Autonomous Region, and northern Guangdong Province in China. This distribution is mainly attributed to the adaptability of *M. officinalis* to the subtropical monsoon climate, including its demand for moderate humidity, ample precipitation, and a preference for loose, fertile, well-drained slightly acidic or neutral soils, and benefits from the rich cultivation experience and traditions in these areas (Yang et al., 2015).

Under the SSP1-2.6 scenario, an increase in the suitable habitat for *M. officinalis* is anticipated in the 2050s, particularly with a significant expansion of moderately suitable habitats. This result is consistent with the research findings on *Magnolia wufengensis* (Shi et al., 2021), indicating that the suitable habitat for species may expand under moderate climate scenarios. However, as climate scenarios become more severe, such as SSP5-8.5, the increase in suitable habitat is expected to diminish, and the reduction in highly suitable habitats will be more pronounced. This may reflect the potential adverse impacts of climate change on the quality of species habitats. These findings emphasize the threat of climate change to biodiversity and the importance of considering future climate change scenarios when developing conservation strategies. Particularly under harsher climate scenarios, such as SSP5-8.5, the increase in suitable habitat for *M. officinalis* is reduced, and the decrease in highly suitable habitats is more significant, which is similar to the research results of Shi et al. (2024) and Zhan et al. (2022). These improvements are crucial for enhancing the model’s predictive accuracy in new areas, especially in research on the impact of climate change on species distribution, providing more reliable predictive outcomes for ecological conservation and species management.

Further analysis reveals that the suitable habitat of *M. officinalis* not only changes in area but also shows a significant trend of migration in spatial distribution. It is predicted that under the SSP3-7.0 and SSP5-8.5 scenarios, the centroid of the suitable habitat for *M. officinalis* will shift towards the northwest, which may be related to changes in temperature and precipitation patterns caused by climate change. This result aligns with other studies showing that rising temperatures prompt species to move towards the northwest of China (Xu et al., 2018; Liu et al., 2021). These findings uncover the adaptive migration strategies that species may adopt in the face of climate change (Li et al., 2021).

### 4.4 Limitations and future research

The study acknowledges certain limitations that could influence the accuracy of the predictions made for the distribution of *M. officinalis* under future climate scenarios. One primary limitation is the reliance on current climate data and models, which may not fully capture the complexities and uncertainties of future climate conditions. Additionally, the MaxEnt model, although optimized, makes certain assumptions about species-environment relationships that may not account for potential changes in species’ adaptability or shifts in ecological interactions. The study also recognizes that it does not consider other biotic factors such as species interactions, disease, or the potential for assisted migration, which could significantly affect the distribution and survival of *M. officinalis*. Furthermore, the absence of occurrence records from Tibet and limited data from Taiwan represent spatial gaps that may affect the generalizability of our predictions for these regions. Future studies should prioritize field surveys in these under-sampled areas to validate model predictions. The projected habitat shifts may be conservative estimates, as our model does not explicitly account for increased frequency and intensity of extreme climate events under warming scenarios. Episodes of extreme cold or drought could create population bottlenecks that accelerate local extinctions beyond our predictions.

While our climate scenarios capture mean temperature and precipitation changes, they may underestimate the impact of extreme weather events. Future research should incorporate indices of climate extremes, such as consecutive dry days and heat wave frequency, which may disproportionately affect *M. officinalis* survival at range margins. For subsequent research, it is advisable to adopt more dynamic models that can better simulate the complex interactions between *M. officinalis* and their biotic environments. Long-term monitoring of *M. officinalis* populations across various habitats would provide empirical data to validate and refine model predictions. It would also be beneficial to explore the genetic diversity of *M. officinalis* to understand its potential to adapt to new climatic conditions. Incorporating local climate models with higher resolution and more nuanced data could enhance the precision of future distribution forecasts. Lastly, considering the role of human-mediated dispersal and conservation efforts in shaping species distribution is essential for developing effective conservation strategies.

## 5 Conclusion

In this study, we employed an optimized MaxEnt model to project the potential distribution of *Magnolia officinalis* under various climate scenarios. The model demonstrated high predictive accuracy with an AUC value of 0.917. Our results indicate that suitable habitats for *M. officinalis* are likely to expand by the 2050s under moderate scenarios like SSP1-2.6 but may contract under more severe conditions such as SSP5-8.5. This underscores the significant impact of climate change on habitat quality and extent. We propose conservation actions such as establishing protected areas in predicted suitable regions, implementing monitoring programs, and considering assisted migration for vulnerable populations. Our findings also highlight the importance of integrating climate projections into conservation planning for similar species and ecosystems affected by climate change, emphasizing the need for adaptive management strategies to ensure biodiversity conservation and sustainable resource use.

Based on our findings, we propose the following conservation actions: 1) Corridor establishment: create ecological corridors connecting fragmented populations in Hubei-Shaanxi border regions to facilitate north-westward migration, prioritizing riparian zones along the Han River system. 2) Priority conservation sites: establish *in situ* conservation reserves in southeastern refugia (northern Fujian, southern Zhejiang) where high-quality habitats face imminent loss, focusing on populations with unique genetic lineages. 3) *Ex situ* conservation network: develop seed banks at three strategic locations (Wuhan Botanical Garden, Kunming Institute of Botany, and South China Botanical Garden) to preserve genetic diversity from across the species’ range. 4) Assisted migration trials: initiate experimental translocations from southeastern populations to climatically suitable sites in Shaanxi Province, monitoring establishment success over 5-year intervals. 5) Community-based conservation: engage local communities in sustainable harvesting programs, establishing cultivation guidelines that maintain 60% of mature trees for seed production.

## Data Availability

The raw data supporting the conclusions of this article will be made available by the authors, without undue reservation.
